# Performance Comparison of In-House and Commercial Biosynex Helmints AMPLIQUICK^®^ Real-Time PCR Assays for the Diagnosis of *Schistosoma mansoni* and *Strongyloides stercoralis* in Stool Samples

**DOI:** 10.3390/diagnostics15222928

**Published:** 2025-11-19

**Authors:** Davide Treggiari, Francesca Tamarozzi, Fabio Formenti, Salvatore Scarso, Barbara Pajola, Lavinia Nicolini, Cristina Mazzi, Francesca Perandin

**Affiliations:** 1 Department of Infectious-Tropical Diseases and Microbiology, IRCCS Sacro Cuore Don Calabria Hospital, 37024 Negrar di Valpolicella, VR, Italy; davide.treggiari@sacrocuore.it (D.T.); fabio.formenti@sacrocuore.it (F.F.); salvatore.scarso@sacrocuore.it (S.S.); barbara.pajola@sacrocuore.it (B.P.); lavinia.nicolini@sacrocuore.it (L.N.); francesca.perandin@sacrocuore.it (F.P.); 2 Clinical Research Unit, IRCCS Sacro Cuore Don Calabria Hospital, 37024 Negrar di Valpolicella, VR, Italy; cristina.mazzi@sacrocuore.it

**Keywords:** *Schistosoma mansoni*, *Strongyloides stercoralis*, stool, molecular diagnosis, RT-PCR

## Abstract

**Background/Objectives**: The timely diagnosis of schistosomiasis and strongyloidiasis is important because of their potentially severe, even lethal, consequences. European diagnostic laboratories must comply with the European In Vitro Diagnostic (IVD) Regulation, which requires justifying the use of in-house assays when CE-IVD-marked kits are available. We aimed to compare the performance of the Biosynex Helminths AMPLIQUICK^®^ RT-PCR and the multiplex in-house RT-PCR for the diagnosis of *Schistosoma mansoni* and *Strongyloides stercoralis* currently used in our department, an Italian reference centre for tropical diseases. **Methods**: We conducted a performance comparison study on biobanked frozen stool samples classified as cases or controls according to PCR and/or copromicroscopy at diagnosis. Both RT-PCRs were performed on DNA re-extracted from the same stool aliquot. Sensitivity and specificity were compared using McNemar’s Chi-squared test, while agreement was assessed using Gwet’s AC1 and Cohen’s K coefficients, and Bland–Altman analysis. **Results**: A total of 45 *S. mansoni* cases with 52 controls and 29 *S. stercoralis* cases with 54 controls were analyzed. For both *S. mansoni* and *S. stercoralis*, sensitivity and specificity were not significantly different between RT-PCRs (*p* = 1). Concordance was perfect for controls (AC1 = 1) in both cohorts, but was poor for *S. mansoni* cases (AC1 = 0.38) and good for *S. stercoralis* cases (AC1 = 0.78). **Conclusions**: Performance was not significantly different between in-house and Biosynex RT-PCRs. Nevertheless, careful assessment of the specific molecular targets included in the panels and prospective evaluation of any newly introduced tests should be implemented to minimize the impact of clinically significant discrepancies.

## 1. Introduction

Schistosomiasis and strongyloidiasis are infections caused by the helminths *Schistosoma* spp. and *Strongyloides stercoralis*, respectively, both listed by the World Health Organization (WHO) among the Neglected Tropical Diseases (NTDs) [1]. Waterborne trematode parasites of the genus *Schistosoma* are endemic in 78 tropical and subtropical countries, affecting over 200 million people, mainly in Africa, and causing about 2.5 million disability-adjusted life years (DALYs) and 2400 deaths annually [1,2,3]. *Schistosoma* parasites cause urogenital (*S. haematobium*) and hepato-intestinal (*S. mansoni*, *S. japonicum*, and other less prevalent species) schistosomiasis. Disease pathogenesis originates from granulomatous inflammation followed by fibrosis around parasite eggs, which are released for years by female worms and remain entrapped in organs and tissues, leading to organ dysfunction, cancer associated with *S. haematobium*, and portal hypertension caused by other species [3]. *S. stercoralis* is a globally distributed soil-transmitted nematode estimated to affect over 600 million people, mainly in South-East Asia, Africa, and the Western Pacific Region [4]. Its peculiar auto-infective cycle can lead to a lifelong infection that may be asymptomatic or cause chronic intestinal, respiratory, and cutaneous symptoms [5]. Immunosuppression can induce fatal hyperinfection/dissemination, in which large numbers of larvae disseminate from the intestine throughout multiple organs [5].

Both parasitic infections are among the most frequently imported NTDs in Europe [6]. However, both infections can remain silent or paucisymptomatic with nonspecific symptoms for years, making their diagnosis difficult and often extremely delayed, especially outside specialized centres in non-endemic areas [7]. Classical diagnostic methods for intestinal schistosomiasis and strongyloidiasis include stool parasitological examination by microscopy and serological testing [8,9]. However, detecting and correctly identifying parasite eggs (*Schistosoma*) and larvae (*S. stercoralis*) in stool might have low sensitivity and require a well-equipped parasitology laboratory with specific expertise in microscopy. Serology may be more easily implemented in non-specialized laboratories, but available serological assays show highly variable sensitivity and specificity, and cannot distinguish between present and past infection (especially in the case of schistosomiasis). Molecular detection of parasite DNA in stool is also possible for diagnosing intestinal helminths [8,9]. Molecular assays generally show no greater sensitivity for helminths than a well-performed coproparasitological examination [8,9] and are relatively costly. However, the operator-independency of these techniques, the general availability of the equipment in diagnostic laboratories in high-resource settings, and the availability of commercial kits, make molecular diagnostics a valuable and appealing option especially in non-endemic areas. This is even more true in the current situation of European diagnostic laboratories having to comply with the In Vitro Diagnostic (IVD) Regulation (IVDR; Regulation (EU) 2017/746 [10]), which requires, among other things, the development and maintenance of detailed documentation of the entire test lifecycle (design, performance, risk management, clinical use, follow-up), and, by 2030, justify the use of in-house assays as opposed to commercially available CE-IVD-marked kits.

In light of the new IVDR regulation, therefore, European diagnostic laboratories must evaluate the performance of their in-house assays and compare them with those of available CE-IVD-marked tests to support the decision of whether to accredit in-house methods already in use or replace them with commercial assays. Because of the peculiarities of NTDs (diagnostic difficulties, comprehensive diagnostic capacity available only in reference centres) and the severe consequences that may derive from a missed or incorrect diagnosis, especially in the case of schistosomiasis and strongyloidiasis, validation of diagnostic assays for these infections for use in clinical settings is imperative. In this context, we compared the performance of the commercial Biosynex Helminths AMPLIQUICK^®^ real-time PCR (RT-PCR) and the in-house RT-PCR for the molecular diagnosis of *S. mansoni* and *S. stercoralis* currently used in the Department of Infectious-Tropical Diseases and Microbiology (DITM) of IRCCS Sacro Cuore Don Calabria Hospital, Negrar, Italy, a reference centre for infectious-tropical diseases located in Northern Italy which hosts the WHO Collaborating Centre on strongyloidiasis and other NTDs. Using selected, archived stool samples, we found no significant differences in performance between the in-house PCR routinely applied at DITM and the commercial Biosynex PCR for *S. mansoni* and *S. stercoralis*.

## 2. Materials and Methods

### 2.1. Study Design

This is a performance comparison study performed on stool samples meeting the eligibility criteria for inclusion (see below) that were stored frozen in the Tropica Biobank (bbmri-eric ID: IT_1605519998080235) of IRCCS Sacro Cuore Don Calabria Hospital.

The study is reported according to the STARD guidelines for reporting diagnostic accuracy studies [11].

### 2.2. Sample Selection and Classification

All stool samples stored in the Tropica Biobank and originally processed for diagnosis of intestinal schistosomiasis or strongyloidiasis based on epidemiological and/or clinical grounds from 2018 onwards were eligible for selection. This date was chosen because of the start of implementation of the current DNA extraction and in-house RT-PCR protocol at DITM. Samples were eligible for inclusion in the study as positive (cases) or negative (controls) according to the following criteria: (1) *Schistosoma* cases: positivity in the in-house RT-PCR and/or presence of *S. mansoni* eggs in stool observed by microscopy after formol-ether concentration technique (FECT) at diagnosis on the same sample; (2) *Schistosoma* controls: negativity in both in-house RT-PCR and coproparasitology (FECT); (3) *Strongyloides* cases: positivity on the in-house RT-PCR and/or presence of *S. stercoralis* larvae in stool (examined after coproculture and/or FECT) at diagnosis on the same sample; (4) *Strongyloides* controls: negativity in both in-house RT-PCR and coproculture at diagnosis on the same sample.

At DITM, routine coproparasitological analysis is performed using FECT, followed by microscopy of the concentrated stool sediment. For the specific search for nematode larvae, stool culture is routinely performed by plating 5 g of stool mixed with charcoal (2:1 ratio) on 10 agar plates for culturing *Strongyloides* (Biolife italiana Srl, Milan, Italy), incubating them at 26 °C for 6 days, and assessing daily for the presence of larvae by stereomicroscopy. Final negativity or morphological identification of any larva present [8] is then defined by rinsing the plate surface and examining the sediment under an optical microscope.

### 2.3. DNA Extraction

DNA was re-extracted from all fecal samples before performing both the in-house and Biosynex RT-PCR assays. For each sample, two frozen 0.5 mL aliquots were thawed, thoroughly mixed, and redivided into two aliquots. One aliquot was processed through the in-house RT-PCR pre-extraction procedure, and the other through the Biosynex RT-PCR pre-extraction procedure. In short, for the in-house method, samples were placed in tubes containing beads (MagnaLyzer Green Beads Roche, Basel, Switzerland) supplemented with 100 µL of S.T.A.R. buffer (Roche, Basel, Switzerland) and 4 µL of internal control (Phocid alphaherpesvirus 1—PhHV-1). The PhHV-1 was added to achieve an approximate threshold cycle (Ct) value between 25 and 30. Samples were centrifuged in the MagnaLyzer (Roche) at 3000× *g* rpm for 30 s, followed by a brief centrifugation at 10,000× *g* rpm for 10 s in a centrifuge with a 20 cm radius rotor. They were then incubated at 95 °C for 10 min in a thermal block, vortexed for 10 s, and centrifuged for 1 min at 10,000× *g* rpm in a centrifuge with a 20 cm radius rotor. The supernatant was collected for DNA extraction.

For the commercial assay, the manufacturer’s instructions were followed. Briefly, stool samples were transferred into a proprietary vial containing beads and pretreatment buffer (BIOSYNEX AMPLIQUICK^®^ Fecal Pretreatment Kit, Illkirch-Graffenstaden, France). For loose stools, 200 µL was pipetted into the vial. Ten µL of procedural control from the Biosynex AMPLIQUICK^®^ kit was added. Samples were mechanically homogenized using a Ruptor Elite Bead Mill (OMNI international, Kennesow, GA, USA; 4 m/s, 2 cycles of 30 s, with a 10 s pause), incubated at 95 °C for 5 min, and centrifuged at 10,000× *g* rpm for 1 min in a 20 cm radius rotor centrifuge. The supernatant was collected for DNA extraction.

After pre-extraction, each sample was processed using the same automated DNA extraction method, which included magnetic bead-based binding of nucleic acids in the presence of lysis/binding buffer, followed by three washing steps to remove inhibitors. Briefly, 150 µL of pre-extracted stool samples was diluted in 450 µL of nuclease-free water with the Hamilton MagEx system (Hamilton Company, Reno, NV, USA). Nucleic acid extraction was performed using the MagMAX™ Viral/Pathogen Nucleic Acid Isolation Kit (Thermo Fisher Scientific, Waltham, MA, USA) according to the manufacturer’s instructions, adapted for automation on the Hamilton MagEx platform. The bound nucleic acids were then eluted at room temperature in 100 µL of elution buffer provided with the kit.

Each RT-PCR (in-house and Biosynex) was run by personnel blinded to the results of the other RT-PCR. However, blinding to the original classification of samples (case or control) based on diagnostic tests performed at diagnosis was not possible, as the same personnel who performed the PCRs also retrieved the selected samples from the biobank.

### 2.4. In-House RT-PCR

The multiplex in-house RT-PCR implemented at DITM is based on Obeng et al. [12] for *Schistosoma* spp., and on Verweij et al. [13,14] for *S. stercoralis* (which also amplifies *S. fuelleborni fuelleborni* [15]). It also includes *Hymenolepis nana*, based on the protocol of Akenten et al. [16]. The internal control, PhHV-1, is detected using primers and a probe as described by Niesters [17]. The nucleotide sequences of the primer and probe systems used for the in-house RT-PCR are available in Table S1. PCR reactions (25 μL) contained 2×SsoFast Mastermix (Bio-Rad, Hercules, CA, USA), 2.5 μg BSA (Sigma-Aldrich, St Louis, MO, USA), 80 nM of each *Schistosoma* and PhHV-1 primer, 100 nM of each *S. stercoralis* and *H. nana* primer, 100 nM of each species-specific probe (200 nM for PhHV-1), and 5 μL of sample DNA. PCR cycling was performed on a CFX Real-Time System (Bio-Rad) under the following conditions: 95 °C for 3 min, followed by 40 cycles of 95 °C for 15 s, 60 °C for 30 s, and 72 °C for 30 s.

Since the Biosynex Helminths AMPLIQUICK^®^ assay also includes other helminth targets (see below), we analyzed the whole panel of samples also with the in-house multiplex RT-PCR for geohelminths currently performed at DITM. However, we could not formally compare the performance of the two RT-PCRs on these further targets using statistical analysis. Targets of the in-house RT-PCR for geohelminths include *Trichuris trichiura*, *Ascaris lumbricoides*, *Ancylostoma duodenale*, and *Necator americanus*, with primers and probes based on the work of Llewellyn et al. [18]. PCR reactions (25 μL) contained 2 × SsoFast Mastermix (Bio-Rad), 2.5 μg BSA (Sigma-Aldrich), and 300 nM of each primer and 80 nM of each probe for *T. trichiura*, *A. duodenale*, and *N. americanus*; 200 nM of each primer and probe for *A. lumbricoides*; 100 nM of the PhHV-1 probe; and 5 μL of DNA sample.

For both RT-PCRs, cycling was performed on a CFX Real-Time System (Bio-Rad) under the following conditions: 95 °C for 3 min, followed by 40 cycles of 95 °C for 15 s, 60 °C for 30 s, and 72 °C for 30 s.

### 2.5. Commercial RT-PCR

The Biosynex Helmints AMPLIQUICK^®^ assay was performed as per the manufacturer’s instructions, using 96-well microplates pre-loaded with ready-to-use master mixes targeting specific parasite DNA. Declared targets include *S. mansoni*, *S. stercoralis* (for both, declared species-specific detection), *A. lumbricoides*, *T. trichiura*, and *Diphyllobothrium latum* (declared detection also of *D. nihonkaiense*) (target COI); *Taenia* spp. (target IGS); *H. nana* (target ITS2); *Enterobius vermicularis* (target 5S rRNA); and *A. duodenale* and *N. americanus* (target ITS1). The nucleotide sequences of the primer and probe systems are not made available by the manufacturer of the commercial assay. Each master mix contained dNTPs, MgCl_2_, fluorescent primers/probes, Taq polymerase, and buffer. An internal control (CIEZ) for monitoring PCR inhibition is also included, as well as a procedural control (IPC) to verify DNA extraction. The internal control should ideally be detected within 30 cycles (Ct ≤ 30).

RT-PCR cycling was performed on a CFX Real-Time System (Bio-Rad) under the following conditions: 95 °C for 3 min followed by 50 cycles of 95 °C for 5 s and 58 °C for 20 s.

### 2.6. RT-PCR Output

The Ct output was analyzed using BioRad CFX Maestro 1.1 software (version 4.1.2433.1219). Amplification signals that exhibited the characteristic morphology of a RT-PCR amplification curve were classified as positive, whereas negativity was defined as the absence of amplification signal; positivity was defined as Ct ≤ 40. Runs failing amplification of internal controls were considered as not determined.

### 2.7. Statistical Analysis

The sample size of the study was limited by the number of eligible samples available in the biobank and, because of financial constraints, by the maximum number of tests made available free of charge by the manufacturer of the commercial assay. These limitations resulted in 45 *Schistosoma* cases and 52 controls, and 29 *Strongyloides* cases and 54 controls meeting eligibility criteria being used in the study (Table 1). Based on the analysis of routine diagnostic results at DITM combining coproparasitological and molecular assays, the sensitivity and specificity of the in-house RT-PCR on stool were calculated as 88% and 99% for *Schistosoma* and 80% and 99% for *Strongyloides*, respectively. Specifically, for *Schistosoma*, of 2800 samples processed with both in-house RT-PCR and FECT between 2008 and 2020, 130 were positive in at least one test: 81 (62%) were positive in both tests, 15 (12%) only in copromicroscopy and 34 (26%) only in RT-PCR. For *Strongyloides*, of 68 samples processed with both in-house RT-PCR and coproculture, 5 were positive in at least one test: 4 (80%) were positive in both tests, 1 (20%) only in coproculture, and none were positive only in RT-PCR. Based on these figures, we estimated that the available sample sizes had 80% power to detect with 5% type I error a difference in sensitivity of 20% for *Schistosoma* and 35% for *Strongyloides* and a difference in specificity of 15% for both *Schistosoma* and *Strongyloides* between the in-house and the Biosynex assays using two-sided McNemar test.

Data were summarized as numbers and percentages and as median and interquartile ranges (IQR) as appropriate. Sensitivity and specificity with 95% confidence intervals (CI) were calculated from 2 × 2 tables. These were then compared between the two RT-PCRs using McNemar’s Chi-squared test. The degree of agreement between the two RT-PCRs was assessed using Gwet’s AC1 coefficient [19] and Cohen’s K coefficient. Bland–Altman analysis was used to calculate the mean bias (“In-house RT-PCR Ct”—“Biosynex RT-PCR Ct”), the standard deviation (SD) of the differences, and the limits of agreement (LoA), defined as mean bias ± 1.96 × SD of the differences. In addition, the 95% CIs of the bias and LoA were estimated. Proportional bias was further evaluated by regressing the differences in Ct values of the in-house RT-PCR Cts across the overall sample using generalized linear regression. All statistical analyses were performed in R software (version 4.5.1).

## 3. Results

All study data are available in File S1. Table 1 summarizes the number of positive samples at diagnosis (in FECT and/or coproculture and in-house RT-PCR) and when the in-house and Biosynex RT-PCRs were newly performed for their comparison.

### 3.1. Characteristics of the Cohort Samples at Diagnosis

Twenty-four of the 45 (53%) available *Schistosoma* case samples were defined at diagnosis by a concordant positivity in both in-house RT-PCR and copromicroscopy after FECT, while of the remaining 21 samples, seven (7/45; 16%) had a positive microscopy but negative RT-PCR, and 14 (14/45; 31%) had positive RT-PCR but negative microscopy. As per inclusion criteria, all 52 *Schistosoma* control samples were defined by the negativity at diagnosis in both in-house RT-PCR and copromicroscopy after FECT.

Of the 29 *Strongyloides* case samples available, all had a positive in-house RT-PCR at diagnosis. Culture was performed on 11 samples at diagnosis, which was positive in seven cases. FECT was performed on 26 samples at diagnosis, being positive in 18 cases. Culture and FECT were performed concurrently at diagnosis only in 11 cases (concordant positive, *n* = 5; culture-positive/FECT-negative, *n* = 2; culture-negative/FECT-positive, *n* = 4). As per inclusion criteria, all 54 *Strongyloides* control samples were defined by the negativity at diagnosis of both in-house RT-PCR and copromicroscopy after culture.

Overall, the distribution of positive results across assays comprising the “cases” groups in this study was not representative of the distribution of positivities in different assays observed in our Department at routine diagnosis, and described above in Section 2.7. Thus, importantly, the sensitivity and specificity values calculated in this study were used solely to compare the performance of the two RT-PCRs and should not be interpreted as absolute measures of diagnostic accuracy.

### 3.2. Comparative Performance of In-House RT-PCR and Biosynex RT-PCR for the Detection of S. mansoni

**Figure 1:**
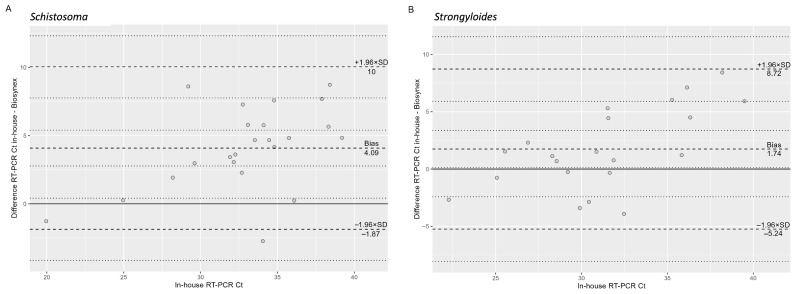
Bland–Altman plot showing the mean bias (“In-house RT-PCR Ct”—“Biosynex RT-PCR Ct”) and the limits of agreement (LoA), defined as mean bias ± 1.96 × standard deviation (SD) of the differences (dashed lines). The 95% CIs of the bias and LoA are indicated by the dotted lines. (**A**) = analysis of RT-PCR results for *S. mansoni*. (**B**) = analysis of RT-PCR results for *S. stercoralis*.

Results of the in-house and Biosynex RT-PCRs for *Schistosoma* performed in parallel after defrosting and processing of samples are summarized in Table 2, while Table 3 shows the performance comparison between the two tests. Among case samples, the result for one sample could not be obtained from either the in-house or the Biosynex RT-PCR because of unsuccessful amplification of the respective internal controls. Among the control samples, the result of one sample could not be determined because of lack of internal control amplification in the in-house RT-PCR, while the result was negative with the Biosynex PCR. Among samples with a positive result, the median (IQR) Ct value of the in-house RT-PCR was 31.6 (29.1–36.1) and of the Biosynex RT-PCR was 31.1 (27.5–33.3). As shown in the Bland–Altman plot (Figure 1A), the absolute difference between the two methods increased significantly with higher Ct values (β = 0.51, *p* = 0.002).

When analyzed against the sample classification at diagnosis, the sensitivity of both the re-run in-house RT-PCR and Biosynex RT-PCR was 59.1% and the specificity 98.0% and 98.1%, respectively. Again, it must be highlighted that these figures should just be considered comparatively between assays, and not in absolute terms. Of note, one control sample resulted positive when re-analyzed using both the in-house and the Biosynex RT-PCR; this accounts for their specificity being calculated at 98%. It was not possible to confirm the true positivity of this result by sequencing; however, considering the positive result as bona fide true, this result should be interpreted as higher sensitivity of the RT-PCRs rather than suboptimal specificity.

Sensitivity and specificity were not statistically significantly different between the two tests (McNemar’s Chi-squared test: *p* = 1). Concordance between tests was perfect for controls (percentage agreement 100%; AC1 coefficient = 1; Cohen’s K = 1). On the contrary, when considering case samples, despite overlapping sensitivity (26/44 cases detected as positive in both assays), the concordance between tests was poor (percentage agreement 68.2%; AC1 coefficient = 0.38 [95% CI 0.094–0.674]; Cohen’s K = 0.19 [95% CI: −0.46–0.84]). Indeed, 7 (15.6%) cases were missed by either of the two RT-PCRs.

### 3.3. Comparative Performance of In-House RT-PCR and Biosynex RT-PCR for the Detection of S. stercoralis

Results of the in-house and Biosynex RT-PCRs for *Strongyloides* performed in parallel after defrosting and processing of samples are summarized in Table 4, while Table 5 shows the performance comparison between the two tests. Internal controls were successfully amplified in all samples. Among samples with a positive result, the median (IQR) Ct value of the in-house RT-PCR was 33.8 (31.9–36.1) and of the Biosynex RT-PCR was 28.9 (26.7–30.4). As shown in the Bland–Altman plot (Figure 1B), the absolute difference between the two methods increased significantly with higher Ct values (β = 0.34, *p* = 0.016).

When analyzed against the sample classification at diagnosis, the sensitivity of the re-run in-house RT-PCR was 89.7% (i.e., 10.3% of samples that were positive at diagnosis in the in-house RT-PCR were no longer detected as positive upon re-analysis) and of the Biosynex RT-PCR was 86.2%. The specificity was 100% for both tests (i.e., no new positivity was detected in the control group). Again, it must be highlighted that these figures should just be considered comparatively between assays, and not in absolute terms. Sensitivity and specificity were not statistically significantly different between the two tests (McNemar’s Chi-squared test: *p* = 1). Concordance between tests was perfect for controls (percentage agreement 100%; AC1 coefficient = 1; Cohen’s K = 1), and good for cases (percentage agreement 82.8%; AC1 coefficient = 0.78 [95% CI 0.565–0.997]; Cohen’s K = 0.19 [95% CI: −0.46–0.84]).

### 3.4. Other Targets

The results of the in-house and Biosynex RT-PCRs for the other targets shared between the two assays (*H. nana*, *A. lumbricoides*, *T. trichiura*, *A. duodenale*, *N. americanus*) are summarized in Table 6. The majority of samples detected as positive belonged to the cohort used to evaluate *Schistosoma* infection (20 detections vs. 1 detection on the *Strongyloides* cohort), and were detected by both assays (12/20 detected by both assays, 1/20 detected only by the in-house RT-PCR, and 7/20 detected only by the Biosynex RT-PCR”). Of note, two *S. stercoralis* cases were detected only by the Biosynex RT-PCR in the “*Schistosoma*” samples cohort and one *Schistosoma* case was detected only by the in-house RT-PCR in the “*Strongyloides*” samples cohort. Unfortunately, no copromicroscopy exam was carried out on this latter sample to identify the infecting *Schistosoma* species, and thus it is impossible to determine whether the Biosynex assay would have missed this case because infection was caused by a species different than *S. mansoni*.

No routine PCR for *E. vermicularis*, *D. latum* and *Taenia* spp. are performed at DITM; therefore, no comparative results can be provided for these targets. No positive results for these targets were obtained by the Biosynex RT-PCR in the sample cohorts investigated in this study.

## 4. Discussion

To comply with the IVDR regulation [10], European diagnostic laboratories need to evaluate the performance of their in-house assays and compare them with those of available CE-IVD-marked tests to decide whether to introduce a commercial assay or accredit in-house methods. Although no strict criteria are provided to compare the in-house and available commercial assays and define their equivalence, it is indicated that the choice of an in-house over a commercially available assay should be made if the latter was not deemed appropriate to respond to the specific technical and clinical requirements of the laboratory/hospital. Thus, accuracy could be interpreted as only one aspect to be considered when comparing the two assays. In this study, we compared the performance of the commercial Biosynex Helmints AMPLIQUICK^®^ and of the in-house RT-PCR used in our Department for the molecular diagnosis of two parasitic infections with potentially lethal consequences, *S. mansoni* and *S. stercoralis*, using characterized biobanked samples.

The results of the head-to-head comparison of sensitivity and specificity of the two RT-PCRs, for both targets, were not statistically significantly different. However, several aspects of these results merit further discussion to frame them correctly.

Firstly, despite PCR being generally considered superior to microscopy for the diagnosis of intestinal parasitic infections, in the specific case of helminth infections this generalization should be applied with caution. The cohorts of samples analyzed in this study were not representative of the distribution of diagnoses obtained in our parasitology laboratory. However, 7/45 (16%) of schistosomiasis cases were microscopy-positive but PCR-negative with either test when the re-analyzed, showing that a relevant proportion of “case” samples are detected only by microscopy, in line with the literature [20]. The same figures could not be calculated for strongyloidiasis since all samples that could be selected for this study were PCR-positive at diagnosis. However, the fact that a proportion of patients with strongyloidiasis is only detected by coproparasitology methods is well known [8]. This highlights the complementarity of microscopy and molecular techniques in the field of diagnostic helminthology and the need for always performing copromicroscopy in addition to PCR to minimize falsely negative diagnoses. Several reasons plausibly explain this situation, including the fact that (i) helminth DNA in stool largely derives from parasite eggs/larvae, and is therefore not homogeneously distributed in the fecal volume examined, making it possible that an egg/larva is present in the fecal volume used for microscopy but not for DNA extraction and vice versa; and (ii) the large starting fecal volume used for copromicroscopic techniques compared to that used for DNA extraction, making the presence of eggs/larvae more probable in the portion of the sample used for the former than for the latter. These factors might also partially explain the discrepant results obtained for the same sample when the in-house RT-PCR was run at diagnosis and re-run for the study and between the in-house RT-PCR and the Biosynex RT-PCR when run in parallel. A detrimental effect of the freezing duration and the freeze–thaw process, although having occurred only once, could also have induced a number of negative results at re-run compared to positivity at diagnosis. Unfortunately, Ct values of the in-house RT-PCR when run at diagnosis were not available; therefore, it is not possible to comment on whether some samples might have tested negative at re-run as the consequence of degradation after thawing of samples with originally very low burden of parasitic elements. Finally, the difference in median Ct values of the two assays could also be partly due to technical differences in their protocols and reagents (e.g., differences in pretreatment buffer composition influencing lysis efficiency and DNA recovery or variations in PCR mix formulations affecting amplification efficiency). Although RT-PCR Ct values cannot be linearly related with the actual parasite burden in the human host, the observation that the absolute difference between the two methods increased significantly with higher Ct values highlights that at low target density in each extracted sample, small Ct differences may result in different positive/negative classification of the same sample, with attending clinical consequences.

Secondly, while infection can be defined with certainty in the presence of a positive direct (microscopy or molecular) exam, exclusion of infection (i.e., the status of “control” sample) is intrinsically uncertain. This is exemplified here by the result of a sample included in the “*Schistosoma* controls” group, and was positive for *Schistosoma* in both in-house and Biosynex RT-PCRs when re-run for this study. While we did not carry out sequencing on this sample, and thus we cannot exclude that the result is a false-positive, we tend not to favour this possibility because of the consistency of the positive result in both PCRs and the fact that all control samples derived from patients with a possible infection (based on clinical and/or epidemiological grounds), thus making a positivity plausible. As a consequence, here the specificity lower than 100% calculated for both RT-PCRs could bona fide be interpreted as higher sensitivity rather than suboptimal specificity.

Thirdly, the high percentage of positivity for *S. stercoralis* larvae on microscopy after FECT in the “*Strongyloides* cases” group could erroneously lead one to conclude that this is an appropriate method for the diagnosis of this parasite. However, this is well known not to be the case [8]. The situation occurring in this study actually derives from the sample selection criteria and the routine diagnostic workflow at DITM. Indeed, when larvae are incidentally identified during a FECT exam, coproculture is not always carried out.

A number of study limitations must also be highlighted, the most important of which are the study design and the sample size and selection.

Firstly, we were obliged to use a pre-determined number of stored samples with limited availability. This implies a number of consequences on sample quality (the freeze–thaw cycle could have caused some degree of sample degradation, which unfortunately we could not quantify because of lack of availability of Ct values at diagnosis, as mentioned above) and lack of representativeness of patients/samples processed routinely in our laboratory (selection bias). Also, the results of the in-house RT-PCR were part of the original classification of “cases” and “controls” groups, resulting in incorporation bias. In addition, the case–control diagnostic accuracy design per se is well known to provide different sensitivity and specificity values compared to when the same assay is used in the “real” setting, generally erring towards higher figures. From a retrospective analysis of diagnostic reports in our Department, sensitivity of the in-house RT-PCR was 88% for *S. mansoni* and 80% for *S. stercoralis*. Here, the very different sensitivity figures obtained could partly derive from the study design and the above-mentioned biases. For example, the presence of a positive in-house RT-PCR result for all *Strongyloides* “cases” samples (incorporation bias) could explain the higher sensitivity of the in-house RT-PCR upon re-test (89.7%) compared to what obtained from the analysis of routine diagnostic reports (80%). Unfortunately, the limitations in samples and assays availability did not allow us to overcome these issues. However, all these aspects highlight the fact that sensitivity and specificity figures in this study should be considered only comparatively between assays, and not in absolute terms for definition of assays’ accuracy, as already highlighted.

Secondly, and very importantly, the limited sample size which could be analyzed did not allow a statistical comparison of the performance of the in-house RT-PCR and the Biosynex RT-PCR within stringent, clinically relevant parameters. In other words, our available sample size allowed identifying as statistically significant only a large difference in sensitivity and specificity between the two PCRs, while the clinically acceptable loss of accuracy would be much smaller, since false negative results could have severe consequences on the health of patients. Also, we could not formally compare the performance of the in-house RT-PCR in use at DITM and the Biosynex RT-PCR on targets different than *S. mansoni* and *S. stercoralis* using panels of samples backed by a statistical rationale. In any case, the raw numeric differences in positive samples identified by the in-house RT-PCR and the Biosynex RT-PCR point towards the assays having similar performances on these targets as well. Regarding the targets themselves, the two assays are not completely overlapping, and this could impact clinical decisions. Indeed, the species-specificity of the Biosynex RT-PCR (*S. mansoni* only and *S. stercoralis* only vs. *Schistosoma* spp. and *Strongyloides* spp. of the in-house RT-PCR), while surely addressing the majority of etiologies of intestinal schistosomiasis and strongyloidiasis infections observed in the current European setting, makes the assay inadequate for the identification of other species (e.g., *Schistosoma japonicum*, *Strongyloides fuelleborni*) and possibly of hybrid species, which have equally severe consequences. This stresses even more the above-mentioned need to consider PCR and microscopy as complementary for the diagnosis of helminth infections, and that microscopy should not be abandoned when PCR is introduced.

Thirdly, further limitations derive from the fact that not all diagnostic techniques are applied routinely at diagnosis in our Department (e.g., 18/29 samples in the “*Strongyloides* cases” had no coproculture performed and 48/54 samples in the “*Strongyloides* controls” had no FECT performed). As a result, a formal evaluation of the diagnostic performance of the RT-PCRs compared to other copromicroscopic diagnostic tests could not be attempted.

Finally, as mentioned above, the “control” classification has only a technical meaning in this context, since the actual infection status of the patient from which the sample derived could not be exhaustively investigated, for example, by reviewing the results of additional samples from the same patient tested within the same time frame (this would have fallen outside the ethics clearance covering this study).

## 5. Conclusions

To conclude, this performance comparison study performed on anonymized biobanked stool samples from patients investigated for schistosomiasis and strongyloidiasis found no significantly different performances between the in-house RT-PCR routinely applied at DITM and the commercial Biosynex RT-PCR for *S. mansoni* and *S. stercoralis*. The European IVDR regulation prescribes to privilege CE-IVD-marked commercial assays over in-house methods when assays are comparable. However, accuracy can be interpreted as only one aspect to be considered in this choice, which should also include, among others, specific diagnostic targets and costs. The limitations of this study and the difference in the targets included in the two RT-PCR panels stress the need for a prospective evaluation in the “real setting” of the accuracy and clinical repercussions of any newly introduced test to minimize loss of sensitivity in detection of infections with potentially very severe consequences.

## Figures and Tables

**Table 1:** Results of FECT and/or coproculture at diagnosis, in-house RT-PCR at diagnosis and at re-run for this study, and Biosynex RT-PCR run for this study. FECT = formol-ether concentration technique. APC = agar plate culture. ND = not determined because of lack of amplification of the internal control. Pos = positive. Neg = negative.

Sample Classification at Diagnosis		Coproparasitology (FECT and/or Coproculture) at Diagnosis			In-House RT-PCR at Diagnosis		In-House RT-PCR at Re-Run for the Study			Biosynex RT-PCR		
Pos (Case)	Neg (Control)	Pos	Neg	Not Performed	Pos	Neg	Pos	Neg	ND	Pos	Neg	ND
*Schistosoma* cohort (*n* = 97)												
45	52	FECT 31	FECT 66	0	38	59	26	68	2	26	69	1
*Strongyloides* cohort (*n* = 83)												
29	54	APC 7 FECT 18	APC 58 FECT 15	APC 18 FECT 50	29	54	26	57	0	25	58	0

**Table 2:** Results of in-house and Biosynex RT-PCRs for the detection of *S. mansoni* on defrosted fecal samples. Accuracy figures should be considered comparatively between assays, not in absolute terms. CI = confidence interval. ND = not determined because of lack of amplification of the internal control.

	Sample Classification at Diagnosis			
Assay	Cases (*n* = 45)	Controls (*n* = 52)	Sensitivity (95% CI)	Specificity (95% CI)
In-house RT-PCR				
Positive	26/44 (59.1%)	1/51 (2.0%)	59.1% (43.3–73.3%)	98.0% (88.2–99.9%)
Negative	18/44 (40.9%)	50/51 (98.0%)		
ND	1/45 (2.2%)	1/52 (2.9%)		
Biosynex RT-PCR				
Positive	26/44 (59.1%)	1/52 (1.9%)	59.1% (43.3–73.3%)	98.1% (88.4–99.9%)
Negative	18/44 (40.9%)	51/52 (98.1%)		
ND	1/45 (2.2%)	0		

**Table 3:** Performance comparison between in-house and Biosynex RT-PCR for the detection of *S. mansoni* on defrosted fecal samples. ND = not determined because of lack of amplification of the internal control.

		CASES at Diagnosis (*n* = 45)			CONTROLS at Diagnosis (*n* = 52)	
		Biosynex RT-PCR				
In-house RT-PCR		Positive	Negative	ND	Positive	Negative
	Positive	19/45 (42.2%)	7/45 (15.6%)	0	1/52 (1.9%)	0
	Negative	7/45 (15.6%)	11/45 (24.4%)	0	0	50/52 (96.2%)
	ND	0	0	1/45 (2.2%)	0	1/52 (1.9%)

**Table 4:** Results of in-house and Biosynex RT-PCRs for the detection of *S. stercoralis* on defrosted fecal samples. Accuracy figures should be considered comparatively between assays, not in absolute terms. CI = confidence interval.

	Sample Classification at Diagnosis			
Assay	Cases (*n* = 29)	Controls (*n* = 54)	Sensitivity (95% CI)	Specificity (95% CI)
In-house RT-PCR				
Positive	26/29 (89.7%)	0/54	89.7% (71.5–97.3%)	100% (91.7–100%)
Negative	3/29 (10.3%)	54/54 (100%)		
Biosynex RT-PCR				
Positive	25/29 (86.2%)	0/54	86.2% (67.4–95.5%)	100% (91.7–100%)
Negative	4/29 (13.8%)	54/54 (100%)		

**Table 5:** Performance comparison between in-house and Biosynex RT-PCRs for the detection of *S. stercoralis* on defrosted fecal samples.

		CASES at Diagnosis (*n* = 29)		CONTROLS at Diagnosis (*n* = 54)	
		Biosynex RT-PCR			
In-house RT-PCR		Positive	Negative	Positive	Negative
	Positive	23/29 (79.3%)	3/29 (10.3%)	0	0
	Negative	2/29 (6.9%)	1/29 (3.4%)	0	54/54 (100%)

**Table 6:** Results of in-house and Biosynex RT-PCRs for the detection of overlapping panels’ helminth targets.

RT-PCR Target	Positive in In-House RT-PCR Only	Positive in Biosynex RT-PCR Only	Positive in Both RT-PCRs
***Schistosoma* Cohort (*n* = 97)**			
*Hymenolepis nana*	0	0	8
*Ascaris lumbricoides*	0	0	1
*Trichuris trichiura*	0	2	0
*Ancylostoma duodenale*	0	0	0
*Necator americanus*	1	3	3
*Strongyloides stercoralis*	0	2	0
***Strongyloides* Cohort (*n* = 83)**			
*Hymenolepis nana*	0	0	0
*Ascaris lumbricoides*	0	0	0
*Trichuris trichiura*	0	0	0
*Ancylostoma duodenale*	0	0	0
*Necator americanus*	0	0	0
*Schistosoma mansoni*	1	0	0

## Data Availability

The original contributions presented in this study are included in the supplementary material, File S1. Further inquiries can be directed to the corresponding author.

## Supplementary Materials

The following supporting information can be downloaded at: https://www.mdpi.com/article/10.3390/diagnostics15222928/s1, Table S1: nucleotide sequences of the primer and probe systems used for the in-house PCR. File S1: original study data.

## Abbreviations

The following abbreviations are used in this manuscript:

NTDs	Neglected Tropical Diseases
IVDR	In Vitro Diagnostics Regulation
RT-PCR	Real-Time Polymerase Chain Reaction
DITM	Department of Infectious Tropical Diseases and Microbiology of the IRCCS Sacroc Cuore Don Calabria Hospital, Negrar di Valpolicella, Verona, Italy
PhHV-1	Phocid alphaherpesvirus 1
Ct	threshold cycle
FECT	Formol-Ether Concentration Technique
APC	Agar Plate Culture
