# Supplementary material for: Performance Comparison of In-House and Commercial Biosynex Helmints AMPLIQUICK® Real-Time PCR Assays for the Diagnosis of Schistosoma mansoni and Strongyloides stercoralis in Stool Samples
Source: Diagnostics (Basel). 2025 Nov 19;15(22):2928. doi: 10.3390/diagnostics15222928 (PMC12650956; doi:10.3390/diagnostics15222928)
Supplement: Supplementary file 1 [file diagnostics-15-02928-s001.zip › Supplementary Table S1_primers and probes.pdf]

Table S1. Nucleotide sequences of the primer and probe systems used for the in-house PCR

Target	Nucleotide sequence	GenBank accession N	Length
Strongyloides spp			
Stro18S-1530F	5′-GAATTCCAAGTAAACGTAAGTCATTAGC-3′	AF279916	101 bp
Stro18S-1630R	5′-TGCCTCTGGATATTGCTCAGTTC-3′		
Stro18S-1586T	Cy5.5-5′-ACACACCGGCCGTCGCTGC-3′-BHQ3		
Schistosoma spp			
Ssp_ITS-48F	5′-GGTCTAGATGACTTGATYGAGATGCT-3′	DQ677661	77 bp
Ssp_ITS-124R	5′-TCCCGAGCGYGTATAATGTCATTA-3′	AF503487	
Ssp78T	FAM-5′-TGGGTTGTGCTCGAGTCGTGGC-3′-BHQ1	U22166	
Hymenolepis nana			
HnITS1-1593F	5′-CATTGTGTACCAAATTGATGATGAGTA-3′	AF461124	88 bp
HnITS1-1680R	5′-CAACTGACAGCATGTTTCGATATG-3′		
Hna_1622T	YY-5′-CGTGTGCGCCTCTGGCTTACCG-3′-BHQ1		
Phocid alphaherpesvirus 1			
PhHV-267s	5′-GGGCGAATCACAGATTGAATC -3′	gβgene (S81228)	89bp
PhHV-337as	5′-GCGGTTCCAAACGTACCAA -3′		
PhHV-305tq	Cy5 -5′-TTTTTATGTGTCCGCCACCATCTGGATC-3′-BHQ2		
